# Serum Ferritin in Dengue Infection

**DOI:** 10.7759/cureus.76103

**Published:** 2024-12-20

**Authors:** Yathukulan S, Sundaresan KT

**Affiliations:** 1 Clinical Medicine, University Medical Unit, Teaching Hospital Batticaloa, Batticaloa, LKA

**Keywords:** biomarker, dengue, hemophagocytic syndrome, hyperferritinemia, prognostic indicator, serum ferritin

## Abstract

Dengue fever (DF), a significant global health issue, particularly impacts tropical and subtropical regions. Elevated serum ferritin levels are increasingly recognized as a biomarker for severe dengue infection. This review examines the role of serum ferritin in diagnosing and prognosticating dengue severity. It highlights how hyperferritinemia correlates with severe clinical outcomes, including dengue hemorrhagic fever (DHF) and dengue shock syndrome (DSS). By integrating serum ferritin measurement into routine clinical practice, early identification and intervention for high-risk patients can be enhanced, potentially improving clinical outcomes. Further research should focus on standardizing ferritin level thresholds and exploring the mechanisms underlying ferritin elevation in dengue.

## Introduction and background

Dengue fever (DF) is a significant health concern worldwide, particularly in tropical and subtropical regions; it is caused by the dengue virus, which is an arbovirus of the *Flavivirus* genus with four serotypes and spread by *Aedes* mosquitoes, leading to millions of cases annually. The World Health Organization (WHO) reports that approximately 390 million dengue infections occur each year, with around 96 million manifesting clinical symptoms [1]. Dengue fever is prevalent in more than 100 countries, affecting almost 3.9 billion individuals globally. Regions such as Southeast Asia, the Americas, Africa, and the Eastern Mediterranean face the highest disease burden [2]. The incidence of dengue has surged in recent years, with outbreaks becoming more frequent and severe. Factors such as climate change, urbanization, and increased global travel have contributed to the widespread distribution and intensification of dengue [2]. Understanding these epidemiological trends is crucial for developing targeted prevention and control strategies. In Sri Lanka, dengue is endemic, with periodic outbreaks. In 2023, there were approximately 88,458 cases and 50 reported deaths [3]. The clinical spectrum of dengue infection is variable from mild dengue fever to dengue hemorrhagic fever (DHF) and dengue shock syndrome (DSS), which are serious forms. These severe forms are characterized by severe thrombocytopenia with major bleeding such as gastrointestinal bleeding and intra-abdominal occult bleeding, plasma leakage resulting in fluid accumulation in the peritoneal cavity and pleural space, acute respiratory distress syndrome, and multiorgan failure mainly fulminant hepatic failure. Certain clinical warning symptoms that help to predict the severity are as follows: bleeding, skin petechiae or ecchymosis rashes, nausea, vomiting, intolerable abdominal pain, and hepatosplenomegaly.

Serum ferritin is a known acute-phase protein and can be used as a marker for several pathological conditions, such as systemic lupus erythematosus, rheumatoid arthritis, cancers, neurodegenerative diseases, cardiovascular diseases, metabolic disease, and various infections, including viral infections such as Covid-19, tuberculosis (TB), malaria, and sepsis [4]. Hyperferritinemia is associated with immune activation and coagulation disturbances. A rise in serum ferritin levels has been observed to have a linear correlation with the severity of the above diseases including dengue infection [5].

## Review

Pathogenesis of severe dengue

The development of severe dengue is a complex process that involves the interaction of the virus with the host's immune system. Critical mechanisms in this process include the activation of T and B cells, the occurrence of a cytokine storm, and changes in endothelial function leading to increased vascular permeability and plasma leakage [5]. The differential targeting of specific vascular beds is likely to trigger the localized vascular hyperpermeability underlying DSS. Elevated serum ferritin levels, a marker of the acute-phase response, are also associated with the severity of the disease and reflect the intense inflammatory reaction [5]. Factors such as antibody-dependent enhancement (ADE) and cross-reactive T-cell responses further exacerbate the disease, highlighting the role of immune-mediated damage. Moreover, genetic factors, such as specific gene expressions, contribute to the variability in disease outcomes [5].

The role of ferritin in iron metabolism and inflammation in dengue

Ferritin, an acute-phase reactant, reacts to inflammation and infection by significantly increasing its levels. The production of ferritin is stimulated by cytokines, specifically interleukin-1 (IL-1) and tumor necrosis factor-alpha (TNF-α), in various cell types, including hepatocytes and macrophages [6]. This increased ferritin helps sequester iron within cells, reducing its availability for pathogens that require iron for growth. In dengue, high serum ferritin levels indicate severe disease and correlate with heightened inflammation and immune activation [7]. Elevated ferritin levels in dengue patients can signify severe disease progression and are associated with other inflammation and endothelial activation markers [7].

Current treatment strategies and challenges

Managing dengue primarily involves supportive care, such as maintaining fluid balance, managing fever, and monitoring for severe disease signs. The most effective way to prevent complications from dengue is through careful clinical management, as there is no specific antiviral treatment available [2]. Nonetheless, managing severe cases, such as DHF and DSS, continues to be a difficult task. One of the key challenges is the inability to identify patients at risk for severe disease due to the scarcity of reliable prognostic markers. The timely recognition of these individuals is essential for effective management. Serum ferritin measurement shows promise as it can provide an early indication of disease severity, potentially guiding more aggressive interventions when necessary [5].

Serum ferritin as a diagnostic and prognostic marker

Serum ferritin levels have become a useful diagnostic tool in dengue fever, especially when standard diagnostic methods, such as nonstructural protein 1 (NS1) antigen and antibody tests, may not be sufficient [8]. During the early stages of dengue infection, the NS1 antigen test is usually positive, while IgM antibodies are detectable from day 6. However, there is a period between the disappearance of the NS1 antigen and the appearance of IgM antibodies when neither test may be positive. In this situation, serum ferritin levels can serve as an alternative marker for dengue infection [7]. The review effectively addresses the growing recognition of serum ferritin as a biomarker in dengue fever. However, it is not done in routine clinical practice, and it is a cost-effective strategy in middle-income countries.

According to research, serum ferritin levels are consistently higher in patients with dengue fever than in those with other bacterial or viral infections, which suggests a greater likelihood of complications [5]. In a study conducted on the Caribbean island of Aruba, ferritin was found to be a reliable clinical marker for differentiating dengue from other febrile illnesses [8]. In dengue virus-infected patients, elevated levels of ferritin indicate an active and highly activated disease state with coagulation disturbances and immune activation [8].

A study conducted in India discovered that a serum ferritin level of 1,291 ng/mL demonstrated an impressive 82.6% sensitivity and a perfect 100% specificity in differentiating dengue fever from other febrile illnesses when the NS1 antigen or IgM antibody was not detectable [5,9]. Furthermore, another study in Thai children with dengue infection found that the median serum ferritin levels were significantly elevated in those with DHF as compared to those with DF. A cutoff level of 1,200 ng/mL displayed an encouraging 81.5%-89.9% sensitivity and a reasonable 36.4%-42.4% specificity in predicting DHF on days 5-7 of the illness [10].

Serum ferritin as a marker of severity

Serum ferritin has been widely recognized as a critical biomarker for predicting the severity of dengue infections. Many studies have shown that elevated levels of ferritin are strongly linked to severe clinical manifestations, including DHF and DSS [7]. A systematic review and meta-analysis revealed that patients with severe dengue had significantly higher serum ferritin levels than those with non-severe dengue, with a standardized mean difference (SMD) of 4.05 (95% CI: 2.09-6.00) [11]. This showed ferritin is a severity marker.

A study conducted in India found that serum ferritin levels were significantly higher in patients with severe dengue compared to those with mild dengue and dengue with warning signs. This study suggested a cutoff value of 714 ng/mL for differentiating severe dengue from non-severe dengue, with a sensitivity of 100% and a specificity of 43.75% [12]. Additionally, a study by Soundravally et al. (2015) found that serum ferritin levels exceeding 1,000 ng/mL were predictive of severe dengue, with sensitivity and specificity values of 87% and 83%, respectively [7]. Furthermore, Chaiyaratana et al. (2008) reported that a ferritin level of 1,200 ng/mL was a reliable predictor of DHF in children, with sensitivities on days 5, 6, and 7 of illness being 81.5%, 84.4%, and 89.9%, respectively [10].

Prognostic value of serum ferritin

Serum ferritin has implications beyond predicting disease severity, as elevated levels also signify poor clinical outcomes. These outcomes include higher hospitalization rates and longer recovery times. A multicenter study involving 500 dengue patients demonstrated that those with ferritin levels above 2,000 ng/mL had a significantly increased risk of prolonged hospitalization (a mean duration of 12 days) compared to those with lower levels (a mean duration of seven days) [13]. Additionally, retrospective analyses of dengue patient data have shown that high ferritin levels are correlated with an increased likelihood of complications, such as liver damage and coagulopathy. In one such study, elevated ferritin levels were associated with a threefold increase in the odds of developing severe liver dysfunction [14].

Research in Aruba and Brazil has demonstrated a strong correlation between elevated levels of serum ferritin and severe dengue virus infection. Elevated ferritin has been connected to a pro-inflammatory cytokine profile, thrombocytopenia, and heightened liver enzyme levels, indicating a significant prognostic value [7]. The results indicate that serum ferritin measurement ought to be included in standard clinical protocols for dengue in order to enhance prognostic accuracy and to guide effective clinical management [5].

In summary, the available data strongly backs the notion that serum ferritin acts as a beneficial prognostic indicator for dengue infection. Specifically, elevated levels of serum ferritin are linked to the severity of the illness and the likelihood of developing life-threatening complications. By incorporating serum ferritin testing into standard clinical practice, healthcare providers can more quickly identify high-risk individuals and implement targeted interventions to improve patient outcomes.

Integrated biomarker assessment in dengue: The role of serum ferritin and hepatic transaminases in predicting disease severity

The use of serum ferritin and liver enzymes together can improve the accuracy of predicting and diagnosing severe dengue infections. Elevated serum ferritin levels, a protein produced by the liver in response to infection and inflammation, are commonly seen in dengue patients, especially in severe cases such as DHF [10]. Simultaneously, dengue fever frequently leads to increased levels of liver enzymes, such as aspartate aminotransferase (AST) and alanine aminotransferase (ALT), which function as indicators of liver involvement and the severity of the disease [15]. Elevated levels of ferritin and hepatic transaminases indicate a significant inflammatory response, which highlights the systemic nature of severe dengue infections [6]. Keeping track of these biomarkers can help identify high-risk patients for severe dengue early on, allowing for timely medical interventions and potentially enhancing clinical outcomes [15]. The utilization of dual biomarker strategies highlights the importance of integrating diagnostic techniques in effectively managing dengue.

Hemophagocytic syndrome (HPS) in dengue and ferritin

Hemophagocytic lymphohistiocytosis (HLH), also referred to as hemophagocytic syndrome (HPS), is a severe inflammatory condition characterized by excessive immune activation and cytokine release. It is often triggered by infections, including dengue. Elevated serum ferritin, a critical biomarker for diagnosing and monitoring this condition, is commonly observed in HPS [5]. In patients with dengue infection, hyperferritinemia is particularly notable in those who develop HPS, reflecting the intense inflammatory response and hemophagocytic activity [16]. The occurrence of HPS in dengue is relatively rare but notable, with studies reporting an incidence rate of 3.6% among hospitalized dengue patients in Taiwan and around 5% in severe dengue cases among pediatric patients in India [16].

The elevated ferritin levels observed in HPS go beyond being a mere byproduct of inflammation. Instead, they are the result of macrophage activation and the subsequent engulfment of erythrocytes, leukocytes, and platelets [17]. This pathological process highlights ferritin's dual role as a marker of iron storage and an indicator of disease severity in dengue-associated HPS [18]. Moreover, the extent of hyperferritinemia in HPS is directly correlated with adverse clinical outcomes, making it a highly valuable prognostic marker [16].

Due to its importance in detecting and predicting severe dengue, measuring serum ferritin in patients can be beneficial in identifying HPS early, which allows for timely and effective clinical interventions [6]. However, it is imperative to differentiate between hyperferritinemia caused by HPS and elevated ferritin levels in other inflammatory conditions, which requires comprehensive clinical and laboratory assessments [18]. By incorporating serum ferritin measurement into the diagnostic framework for dengue patients, healthcare providers can significantly improve the management and prognosis of those at risk of developing HPS [18].

## Conclusions

In conclusion, elevated serum ferritin levels serve as a key biomarker for detecting and predicting the severity of dengue infection. The data indicates that serum ferritin aids in differentiating between mild and severe forms of dengue, providing crucial information for prompt intervention. The persistent correlation between high ferritin levels and severe clinical outcomes emphasizes its potential usefulness in clinical practice. Hence, incorporating ferritin measurement into standard dengue management protocols could improve patient care and results. However, further discussion on the logistical aspects of implementing this in resource-limited settings, such as cost, the availability of tests, and integration with current diagnostic practices, would be important.

## References

[1] (2024). Dengue guidelines, for diagnosis, treatment, prevention and control. https://www.who.int/publications/i/item/9789241547871.

[2] (2024). Dengue - global situation. https://www.who.int/emergencies/disease-outbreak-news/item/2023-DON498.

[3] (2024). The National Dengue Control Unit. https://www.dengue.health.gov.lk/web/index.php/en/.

[4] Vanarsa K, Ye Y, Han J, Xie C, Mohan C, Wu T (2012). Inflammation associated anemia and ferritin as disease markers in SLE. Arthritis Res Ther.

[5] Roy Chaudhuri S, Bhattacharya S, Chakraborty M, Bhattacharjee K (2017). Serum ferritin: a backstage weapon in diagnosis of dengue fever. Interdiscip Perspect Infect Dis.

[6] Kernan KF, Carcillo JA (2017). Hyperferritinemia and inflammation. Int Immunol.

[7] Soundravally R, Agieshkumar B, Daisy M, Sherin J, Cleetus CC (2015). Ferritin levels predict severe dengue. Infection.

[8] van de Weg CA, Huits RM, Pannuti CS (2014). Hyperferritinaemia in dengue virus infected patients is associated with immune activation and coagulation disturbances. PLoS Negl Trop Dis.

[9] Ray U, Dutta S, Mondal S, Bandyopadhyay S (2017). Severe dengue due to secondary hemophagocytic lymphohistiocytosis: a case study. IDCases.

[10] Chaiyaratana W, Chuansumrit A, Atamasirikul K, Tangnararatchakit K (2008). Serum ferritin levels in children with dengue infection. Southeast Asian J Trop Med Public Health.

[11] Shukla S, Jadhav SM, Gurav YK, Parashar D, Alagarasu K (2023). Serum ferritin level as a prognostic biomarker for predicting dengue disease severity: A systematic review and meta-analysis. Rev Med Virol.

[12] Suresh SC, Hanumanthaiah R, Ramakrishna C (2020). Serum ferritin as a prognostic indicator in adult dengue patients. Am J Trop Med Hyg.

[13] Bharathi GK, Aswinth R, Sudarshan S (2019). An observational study on serum ferritin level in dengue fever and its correlation with severity of dengue fever. J Med Sci Clin Res.

[14] Valero N, Mosquera J, Torres M (2019). Increased serum ferritin and interleukin-18 levels in children with dengue. Braz J Microbiol.

[15] Reddy L, Kadambi P, Aashiq SM, Suresh P (2020). Comparative study of serum ferritin levels and hepatic transaminases between uncomplicated paediatric dengue inpatients and other febrile illnesses in Kanchipuram, India. Int J Contemp Pediatr.

[16] Ramos-Casals M, Brito-Zerón P, López-Guillermo A, Khamashta MA, Bosch X (2014). Adult haemophagocytic syndrome. Lancet.

[17] Jordan MB, Allen CE, Weitzman S, Filipovich AH, McClain KL (2011). How I treat hemophagocytic lymphohistiocytosis. Blood.

[18] La Rosée P, Horne A, Hines M (2019). Recommendations for the management of hemophagocytic lymphohistiocytosis in adults. Blood.

